# Evolution of the insect Hox gene cluster: Comparative analysis across 243 species

**DOI:** 10.1016/j.semcdb.2022.11.010

**Published:** 2024

**Authors:** Peter O. Mulhair, Peter W.H. Holland

**Affiliations:** Department of Biology, University of Oxford, 11a Mansfield Road, Oxford OX1 3SZ, UK

**Keywords:** Homeobox, Hexapoda, Gene cluster, Shx

## Abstract

The Hox gene cluster is an iconic example of evolutionary conservation between divergent animal lineages, providing evidence for ancient similarities in the genetic control of embryonic development. However, there are differences between taxa in gene order, gene number and genomic organisation implying conservation is not absolute. There are also examples of radical functional change of Hox genes; for example, the *ftz*, *zen* and *bcd* genes in insects play roles in segmentation, extraembryonic membrane formation and body polarity, rather than specification of anteroposterior position. There have been detailed descriptions of Hox genes and Hox gene clusters in several insect species, including important model systems, but a large-scale overview has been lacking. Here we extend these studies using the publicly-available complete genome sequences of 243 insect species from 13 orders. We show that the insect Hox cluster is characterised by large intergenic distances, consistently extreme in Odonata, Orthoptera, Hemiptera and Trichoptera, and always larger between the ‘posterior’ Hox genes. We find duplications of *ftz* and *zen* in many species and multiple independent cluster breaks, although certain modules of neighbouring genes are rarely broken apart suggesting some organisational constraints. As more high-quality genomes are obtained, a challenge will be to relate structural genomic changes to phenotypic change across insect phylogeny.

## 1 Introduction

Insects display an astounding range of developmental and morphological diversity. Comprising over half of all described animal species, insect diversity has been attributed to high rates of speciation and adaptive radiation in association with flowering plant diversification, underpinned by dynamic rates of gene and genome evolution. Together with the orders Protura, Diplura and Collembola, insects make up the Hexapoda, a clade within Arthropoda consisting of six-legged, mostly terrestrial species. Within Hexapoda there have been several major evolutionary transitions associated with novel phenotypic traits. The evolution of insect wings is one such event which resulted in diversification of body forms within the clade Pterygota [1]. A later event was the emergence of complete metamorphosis in the holometabolous insects, thought to have permitted rapid diversification. Indeed, the most diverse and speciose insect orders are found within the holometabolous pterygotes (Hymenoptera, Coleoptera, Diptera and Lepidoptera). While the insect body plan is generally well conserved, a myriad of morphological novelties have emerged through insect radiation, ranging from pronotal horns on some beetles, sucking mouthparts in Hemiptera and (most) Lepidoptera, stings in bees and wasps, and halteres in Diptera and Strepsiptera.

Changes in developmental processes underlie morphological diversity, and ultimately these developmental changes must be underpinned by inherited genetic changes. Identifying these genetic changes is one of the goals of evolutionary developmental biology (evo-devo) although this is a difficult task when the morphological transitions occurred tens or hundreds of millions of years ago. One place to start is with the genes shared between taxa, and with key roles in development: a set of genes sometimes called the developmental toolkit. The Hox genes are examples of such core developmental genes, encoding position along the anteroposterior axis of most animal embryos. Furthermore, Hox genes code for transcription factors that activate and repress cascades of downstream genes to sculpt the morphology appropriate to that position. Later in development, Hox genes also orchestrate cell differentiation decisions, primarily though not exclusively within their original embryonic expression domains [2]. Changes in the content, order and expression domains of these genes have been implicated in a huge range of morphological novelties in the arthropod body plan [2].

The insect Hox cluster is thought to have consisted ancestrally of 10 genes: *labial (lab)*, *proboscipedia (pb)*, *zerknüllt (zen)*, *Deformed (Dfd)*, *Sex combs reduced (Scr)*, *fushi tarazu (ftz)*, *Antennapedia (Antp)*, *Ultrabithorax (Ubx)*, *abdominal-A (abdA)*, and *Abdominal-B (AbdB)*, similar to that of the bilaterian ancestor [3], [4]. Of these, *zen* and *ftz* have ‘altered’ roles, having switched from their ancestral roles in anteroposterior position specification to extraembryonic membrane patterning (*zen*) and segmentation (*ftz*). Until recently, we have lacked a thorough knowledge of the multiple evolutionary paths that have been taken from this ancestral state across the diversity of insect orders. Here, we provide an updated view of the evolution of the Hox gene cluster across the largest sample of insect genomes sampled to date. We focus on the evolution of Hox cluster organisation, and do not discuss recent work on Hox gene regulation or the changing downstream functions of Hox genes, such as co-option of Hox genes to accessory roles in different orders (e.g. [5], [6], [7], [8], [9]). Furthermore, we focus on protein-coding loci within Hox clusters, and do not cover non-coding RNAs since these cannot be predicted reliably from genome sequences. There is certainly good evidence for antisense lncRNAs produced from within the Hox cluster of insects and other arthropods, as well as annelids, but comparative data are sparse (for review see [10]). We do not report new sequence data here: these analyses are based on publicly-available complete genome sequences, interpreted in the light of previous analyses.

## 2 Hox genes in a new era of insect genomics

In the pre-genomic era of molecular biology, from the early 1980s to around 2005, Hox gene clusters were analysed either by painstaking positional cloning of mutants or by cross- hybridisation of probes to genomic libraries followed by laborious genomic walking and clone-by-clone sequencing, sometimes coupled with in situ hybridization to chromosome spreads [11], [12]. These heroic efforts were limited to a few species, but they started to generate a picture of comparative stasis of insect Hox gene clusters. For example, in the fruitfly *Drosophila melanogaster*, the insect in which Hox genes were first studied, it was clear that the *zen* gene had undergone tandem duplication to give three genes: *zen*, *z2* and *bcd*. Otherwise, there were no examples of Hox gene duplication in that evolutionary lineage. Similarly, in the red flour beetle *Tribolium castaneum*, there is a *zen* duplication to give two genes, but otherwise the cluster is unaltered [13], [14]. It was, of course, clear from the earliest days that splitting of the cluster was possible, as evidenced by the cluster split in *Drosophila melanogaster* with a 9.6 Mb gap between *Ubx* and *Antp*. It was also clear that intergenic distances could be very large, as in *Drosophila melanogaster* and the locust *Schistocerca gregaria* [11]. Nonetheless, these are relatively minor changes compared to whole Hox gene cluster duplication in vertebrates and extensive posterior Hox gene duplication in vertebrates and amphioxus [15], [16].

From the mid-2000s onwards, complete genome sequencing began to be applied to single species, or small sets of related species, and the evolutionary picture was refined. First, it became clear that splitting of the cluster was not a unique event in *Drosophila melanogaster*, as there have been independent splits at different positions in some other *Drosophila* species [17]. Second, lability of the *zen* gene, in terms of propensity to duplication, was reinforced by a remarkable discovery in the genome of the silk moth *Bombyx mori* [18]. This analysis revealed extensive tandem gene duplication, generating at least 13 copies of *zen*: one locus with an amino acid sequence similar to the ancestral *zen* gene, and 12 that have diverged extensively and given the name Shx (Special homeobox) genes. Later analysis of a refined genome assembly suggested the number may be even greater: *zen* plus 15 Shx loci, although not all can encode functional proteins [19]. Genome sequences of two butterflies (*Heliconius melpomene* and the Monarch *Danaus plexippus*) revealed presence of four Shx genes [20], [21], as did low coverage genome skims of the Comma butterfly *Polygonia c-album*, Speckled Wood butterfly *Pararge aegeria*, Scarlet Tiger moth *Callimorpha dominula* and Horse Chestnut leaf-miner *Cameraria ohridella* [19].

The above historical perspective is one of a gradually unfolding picture emerging in a piecemeal manner as each additional genome is sequenced or analysed. But the landscape is now changing rapidly. In 2018, the Earth BioGenome Project was announced, as a bold vision to determine the complete genome sequence of all living eukaryote species [22]. This vision has galvanised action from over 40 affiliated projects, each attempting to determine high quality genome sequences at scale [23]. Among these, the project that has generated the largest number of high quality insect genomes to date is the Wellcome Trust-funded ‘Darwin Tree of Life’ (DToL), focussed on species living in Britain and Ireland [24]. Since 2019, the DToL project has generated 381 complete genome assemblies with over 2000 more species in the genome sequencing pipeline (data as of July 2022: https://portal.darwintreeoflife.org/tracking). These genome assemblies have been determined using long-read DNA sequencing technology (primarily PacBio HiFi) and scaffolding to chromosome-level using Hi-C. As such, they surpass in quality the large majority of genome assemblies previously available. In particular, the large contig sizes scaffolded to chromosomes provides opportunity to determine gene order and distances. Importantly, all data from the DToL project are released openly.

In reviewing the evolution of Hox gene clusters, we consider that the landscape of the field has changed so remarkably in the past two years that we cannot draw conclusions solely from previously published analyses. Instead, we supplement previous findings with analyses of the openly released data from DToL and other genome sequencing projects. We do not report new experimental data here, but rather draw new conclusions from available data. We use these data to summarise patterns of Hox gene duplication and the changes to genomic organisation across insects, using genomic data from 243 species representing 13 insect orders, plus one order of non-insect hexapod as an outgroup (Table 1). We show that insects continue to be an important model for understanding Hox gene evolution and, with the development of further methods and models for genetic manipulation from a phylogenetically diverse set of orders, will be vital for progress in the field of evolutionary developmental biology [25].

**Table 1:** Order, species and genomes used in this study.

Order	Species	Genome
Trichoptera	Limnephilus lunatus	GCA_917563855.1_iiLimLuna2.1_genomic
Trichoptera	Limnephilus marmoratus	GCA_917880885.1_iiLimMarm1.1_genomic
Trichoptera	Limnephilus rhombicus	GCA_929108145.1_iiLimRhom1.1_genomic
Trichoptera	Glyphotaelius pellucidus	GCA_936435175.1_iiGlyPell1.1_genomic
Trichoptera	Eubasilissa regina	GCA_022840565_Eubasilissa_regina
Phasmatodea	Timema cristinae	GCA_002926335_ipTimCris1
Plecoptera	Nemoura dubitans	GCA_921293005.1_ipNemDubi1.1_genomic
Plecoptera	Nemurella pictetii	GCA_921293315.1_ipNemPict2.1_genomic
Plecoptera	Brachyptera putata	GCA_907164805.1_ipBraPut3m.1_genomic
Coleoptera	Pyrochroa serraticornis	GCA_905333025.1_icPyrSerr1.1_genomic
Coleoptera	Rhagonycha fulva	GCA_905340355.1_icRhaFulv1.1_genomic
Coleoptera	Coccinella septempunctata	GCA_907165205.1_icCocSept1.1_genomic
Coleoptera	Malachius bipustulatus	GCA_910589415.1_icMalBipu1.1_genomic
Coleoptera	Adalia bipunctata	GCA_910592335.1_icAdaBipu1.1_genomic
Coleoptera	Ocypus olens	GCA_910593695.1_icOcyOlen1.1_genomic
Coleoptera	Cantharis rustica	GCA_911387805.1_icCanRust1.1_genomic
Coleoptera	Harmonia axyridis	GCA_914767665.1_icHarAxyr1.1_genomic
Coleoptera	Apoderus coryli	GCA_911728435.1_icApoCory1.1_genomic
Coleoptera	Pterostichus madidus	GCA_911728475.1_icPteMadi1.1_genomic
Coleoptera	Agrypnus murinus	GCA_929113105.1_icAgrMuri1.1_genomic
Coleoptera	Podabrus alpinus	GCA_932274525.1_icPodAlpi1.1_genomic
Coleoptera	Philonthus cognatus	GCA_932526585.1_icPhiCogn1.1_genomic
Coleoptera	Leistus spinibarbis	GCA_933228885.1_icLeiSpin1.1_genomic
Coleoptera	Polydrusus cervinus	GCA_935413205.1_icPolCerv1.1_genomic
Coleoptera	Melolontha melolontha	GCA_935421215.1_icMelMelo1.1_genomic
Coleoptera	Rutpela maculata	GCA_936432065.1_icLepMacu1.1_genomic
Coleoptera	Halyzia sedecimguttata	GCA_937662695.1_icHalSede1.1_genomic
Coleoptera	Ophonus ardosiacus	GCA_943142095.1_icOphArdo1.1_genomic
Lepidoptera	Micropterix aruncella	GCA_944548615.1_ilMicArun2.1_genomic
Lepidoptera	Autographa gamma	GCA_905146925.1_ilAutGamm1.1_genomic
Lepidoptera	Laspeyria flexula	GCA_905147015.1_ilLasFlex1.1_genomic
Lepidoptera	Inachis io	GCA_905147045.1_ilAglIoxx1.1_genomic
Lepidoptera	Pieris brassicae	GCA_905147105.1_ilPieBrab1.1_genomic
Lepidoptera	Blastobasis lacticolella	GCA_905147135.1_ilBlaLact1.1_genomic
Lepidoptera	Nymphalis urticae	GCA_905147175.1_ilAglUrti1.1_genomic
Lepidoptera	Euproctis similis	GCA_905147225.1_ilEupSimi1.1_genomic
Lepidoptera	Erynnis tages	GCA_905147235.1_ilEryTage1.1_genomic
Lepidoptera	Hypena proboscidalis	GCA_905147285.1_ilHypProb1.1_genomic
Lepidoptera	Mythimna impura	GCA_905147345.1_ilMytImpu1.1_genomic
Lepidoptera	Apotomis turbidana	GCA_905147355.1_ilApoTurb1.1_genomic
Lepidoptera	Aricia agestis	GCA_905147365.1_ilAriAges1.1_genomic
Lepidoptera	Hylaea fasciaria	GCA_905147375.1_ilHylFasc1.1_genomic
Lepidoptera	Limenitis camilla	GCA_905147385.1_ilLimCami1.1_genomic
Lepidoptera	Xestia xanthographa	GCA_905147715.1_ilXesXant1.1_genomic
Lepidoptera	Phlogophora meticulosa	GCA_905147745.1_ilPhlMeti2.1_genomic
Lepidoptera	Thyatira batis	GCA_905147785.1_ilThyBati1.1_genomic
Lepidoptera	Pieris rapae	GCA_905147795.1_ilPieRapa1.1_genomic
Lepidoptera	Phalera bucephala	GCA_905147815.1_ilPhaBuce1.1_genomic
Lepidoptera	Endotricha flammealis	GCA_905163395.1_ilEndFlam1.1_genomic
Lepidoptera	Noctua fimbriata	GCA_905163415.1_ilNocFimb1.1_genomic
Lepidoptera	Mamestra brassicae	GCA_905163435.1_ilMamBras1.1_genomic
Lepidoptera	Pararge aegeria	GCA_905163445.1_ilParAegt1.1_genomic
Lepidoptera	Craniophora ligustri	GCA_905163465.1_ilCraLigu1.1_genomic
Lepidoptera	Cosmia trapezina	GCA_905163495.1_ilCosTrap1.1_genomic
Lepidoptera	Lymantria monacha	GCA_905163515.1_ilLymMona1.1_genomic
Lepidoptera	Notocelia uddmanniana	GCA_905163555.1_ilNotUddm1.1_genomic
Lepidoptera	Celastrina argiolus	GCA_905187575.1_ilCelArgi3.1_genomic
Lepidoptera	Cyaniris semiargus	GCA_905187585.1_ilCyaSemi1.1_genomic
Lepidoptera	Colias croceus	GCA_905220415.1_ilColCroc2.1_genomic
Lepidoptera	Amphipyra tragopoginis	GCA_905220435.1_ilAmpTrag2.1_genomic
Lepidoptera	Deilephila porcellus	GCA_905220455.1_ilDeiPorc1.1_genomic
Lepidoptera	Ennomos fuscantarius	GCA_905220475.1_ilEnnFusc2.1_genomic
Lepidoptera	Laothoe populi	GCA_905220505.1_ilLaoPopu1.1_genomic
Lepidoptera	Lysandra coridon	GCA_905220515.1_ilLysCori1.1_genomic
Lepidoptera	Mellicta athalia	GCA_905220545.1_ilMelAtha1.1_genomic
Lepidoptera	Melitaea cinxia	GCA_905220565.1_ilMelCinx1.1_genomic
Lepidoptera	Nymphalis polychloros	GCA_905220585.1_ilNymPoly1.1_genomic
Lepidoptera	Spilosoma lubricipeda	GCA_905220595.1_ilSpiLubr1.1_genomic
Lepidoptera	Tinea trinotella	GCA_905220615.1_ilTinTrin1.1_genomic
Lepidoptera	Boloria selene	GCA_905231865.2_ilBolSele5.2_genomic
Lepidoptera	Pieris napi	GCA_905231885.1_ilPieNapi4.1_genomic
Lepidoptera	Vanessa atalanta	GCA_905147765.1_ilVanAtal1.1_genomic
Lepidoptera	Notodonta dromedarius	GCA_905147325.1_ilNotDrom1.1_genomic
Lepidoptera	Vanessa cardui	GCA_905220365.1_ilVanCard2.1_genomic
Lepidoptera	Hecatera dysodea	GCA_905332915.1_ilHecDyso1.1_genomic
Lepidoptera	Mimas tiliae	GCA_905332985.1_ilMimTili1.1_genomic
Lepidoptera	Lycaena phlaeas	GCA_905333005.1_ilLycPhla1.1_genomic
Lepidoptera	Lysandra bellargus	GCA_905333045.1_ilLysBell1.1_genomic
Lepidoptera	Maniola jurtina	GCA_905333055.1_ilManJurt1.1_genomic
Lepidoptera	Pheosia tremula	GCA_905333125.1_ilPheTrem1.1_genomic
Lepidoptera	Abrostola tripartita	GCA_905340225.1_ilAbrTrip1.1_genomic
Lepidoptera	Noctua pronuba	GCA_905220335.1_ilNocPron1.1_genomic
Lepidoptera	Atethmia centrago	GCA_905333075.2_ilAteCent1.2_genomic
Lepidoptera	Glaucopsyche alexis	GCA_905404095.1_ilGlaAlex1.1_genomic
Lepidoptera	Pheosia gnoma	GCA_905404115.1_ilPheGnom1.1_genomic
Lepidoptera	Hesperia comma	GCA_905404135.1_ilHesComm1.1_genomic
Lepidoptera	Biston betularia	GCA_905404145.1_ilBisBetu1.1_genomic
Lepidoptera	Plebejus argus	GCA_905404155.1_ilPleArgu1.1_genomic
Lepidoptera	Anthocharis cardamines	GCA_905404175.1_ilAntCard3.1_genomic
Lepidoptera	Fabriciana adippe	GCA_905404265.1_ilFabAdip1.1_genomic
Lepidoptera	Hedya salicella	GCA_905404275.1_ilHedSali1.1_genomic
Lepidoptera	Erannis defoliaria	GCA_905404285.1_ilEraDefo1.1_genomic
Lepidoptera	Ochlodes sylvanus	GCA_905404295.1_ilOchSylv3.1_genomic
Lepidoptera	Leptidea sinapis	GCA_905404315.1_ilLepSina1.1_genomic
Lepidoptera	Autographa pulchrina	GCA_905475315.1_ilAutPulc1.1_genomic
Lepidoptera	Clostera curtula	GCA_905475355.1_ilCloCurt1.1_genomic
Lepidoptera	Schrankia costaestrigalis	GCA_905475405.1_ilSchCost1.1_genomic
Lepidoptera	Ochropleura plecta	GCA_905475445.1_ilOchPlec1.1_genomic
Lepidoptera	Zeuzera pyrina	GCA_907165235.1_ilZeuPyri1.1_genomic
Lepidoptera	Habrosyne pyritoides	GCA_907165245.1_ilHabPyri1.1_genomic
Lepidoptera	Zygaena filipendulae	GCA_907165275.1_ilZygFili1.1_genomic
Lepidoptera	Crocallis elinguaria	GCA_907269065.1_ilCroElin1.1_genomic
Lepidoptera	Idaea aversata	GCA_907269075.1_ilIdaAver1.1_genomic
Lepidoptera	Blastobasis adustella	GCA_907269095.1_ilBlaAdus2.1_genomic
Lepidoptera	Mythimna ferrago	GCA_910589285.1_ilMytFerr1.1_genomic
Lepidoptera	Noctua janthe	GCA_910589295.1_ilNocJant1.1_genomic
Lepidoptera	Bembecia ichneumoniformis	GCA_910589475.1_ilBemIchn1.1_genomic
Lepidoptera	Ennomos quercinarius	GCA_910589525.1_ilEnnQuei1.1_genomic
Lepidoptera	Carcina quercana	GCA_910589575.1_ilCarQuer1.1_genomic
Lepidoptera	Chrysoteuchia culmella	GCA_910589605.1_ilChrCulm1.1_genomic
Lepidoptera	Tinea semifulvella	GCA_910589645.1_ilTinSemi1.1_genomic
Lepidoptera	Acronicta aceris	GCA_910591435.1_ilAcrAcer1.1_genomic
Lepidoptera	Cydia splendana	GCA_910591565.1_ilCydSple1.1_genomic
Lepidoptera	Ypsolopha scabrella	GCA_910592155.1_ilYpsScab1.1_genomic
Lepidoptera	Amphipyra berbera	GCA_910594945.1_ilAmpBerb1.1_genomic
Lepidoptera	Parapoynx stratiotata	GCA_910589355.1_ilParStra1.1_genomic
Lepidoptera	Pyrgus malvae	GCA_911387765.1_ilPyrMalv3.1_genomic
Lepidoptera	Thymelicus sylvestris	GCA_911387775.1_ilThySylv1.1_genomic
Lepidoptera	Apamea monoglypha	GCA_911387795.1_ilApaMono1.1_genomic
Lepidoptera	Neomicropteryx cornuta	GCA_020383195.1_ilNeoCorn1.1_genomic
Lepidoptera	Hemaris fuciformis	GCA_907164795.1_ilHemFuc1.1_genomic
Lepidoptera	Papilio machaon	GCA_912999745.1_ilPapMach1.1_genomic
Lepidoptera	Sesia apiformis	GCA_914767545.1_ilSesApif2.1_genomic
Lepidoptera	Hydraecia micacea	GCA_914767645.1_ilHydMica1.1_genomic
Lepidoptera	Ptilodon capucinus	GCA_914767695.1_ilPtiCapc1.1_genomic
Lepidoptera	Agrochola circellaris	GCA_914767755.1_ilAgrCirc1.1_genomic
Lepidoptera	Eupsilia transversa	GCA_914767815.1_ilEupTran1.1_genomic
Lepidoptera	Agriopis aurantiaria	GCA_914767915.1_ilAgrAura1.1_genomic
Lepidoptera	Eilema depressum	GCA_914767945.1_ilEilDepe1.1_genomic
Lepidoptera	Eilema sororculum	GCA_914829495.1_ilEilSoro1.1_genomic
Lepidoptera	Spilarctia lutea	GCA_916048165.1_ilSpiLutu1.1_genomic
Lepidoptera	Griposia aprilina	GCA_916610205.1_ilGriApri1.1_genomic
Lepidoptera	Omphaloscelis lunosa	GCA_916610215.1_ilOmpLuno1.1_genomic
Lepidoptera	Mesoligia furuncula	GCA_916614155.1_ilMesFuru1.1_genomic
Lepidoptera	Xestia c-nigrum	GCA_916618015.1_ilXesCnig1.1_genomic
Lepidoptera	Emmelina monodactyla	GCA_916618145.1_ilEmmMono1.1_genomic
Lepidoptera	Agrochola macilenta	GCA_916701695.1_ilAgrMaci1.1_genomic
Lepidoptera	Orgyia antiqua	GCA_916999025.1_ilOrgAnti1.1_genomic
Lepidoptera	Erebia ligea	GCA_917051295.1_ilEreLige1.1_genomic
Lepidoptera	Dryobotodes eremita	GCA_917490735.1_ilDryErem1.1_genomic
Lepidoptera	Selenia dentaria	GCA_917880725.1_ilSelDent1.1_genomic
Lepidoptera	Synanthedon vespiformis	GCA_918317495.1_ilSynVesp1.1_genomic
Lepidoptera	Notodonta ziczac	GCA_918843915.1_ilNotZicz1.1_genomic
Lepidoptera	Eulithis prunata	GCA_918843925.1_ilEulPrun1.1_genomic
Lepidoptera	Philereme vetulata	GCA_918857605.1_ilPhiVetu1.1_genomic
Lepidoptera	Melanargia galathea	GCA_920104075.1_ilMelGala2.1_genomic
Lepidoptera	Furcula furcula	GCA_911728495.1_ilFurFurc1.1_genomic
Lepidoptera	Peribatodes rhomboidaria	GCA_911728515.1_ilPerRhom1.1_genomic
Lepidoptera	Pammene fasciana	GCA_911728535.1_ilPamFasc1.1_genomic
Lepidoptera	Aporia crataegi	GCA_912999735.1_ilApoCrat1.1_genomic
Lepidoptera	Hydriomena furcata	GCA_912999785.1_ilHydFurc1.1_genomic
Lepidoptera	Campaea margaritaria	GCA_912999815.1_ilCamMarg1.1_genomic
Thysanoptera	Thrips palmi	GCF_012932325_itThrPalm1
Collembola	Folsomia candida	GCF_002217175_hcFolCand1
Hymenoptera	Bombus hortorum	GCA_905332935.1_iyBomHort1.1_genomic
Hymenoptera	Bombus pascuorum	GCA_905332965.1_iyBomPasc1.1_genomic
Hymenoptera	Bombus campestris	GCA_905333015.1_iyBomCamp1.1_genomic
Hymenoptera	Vespula germanica	GCA_905340365.1_iyVesGerm1.1_genomic
Hymenoptera	Vespula vulgaris	GCA_905475345.1_iyVesVulg1.1_genomic
Hymenoptera	Nomada fabriciana	GCA_907165295.1_iyNomFabr1.1_genomic
Hymenoptera	Vespa crabro	GCA_910589235.1_iyVesCrab1.1_genomic
Hymenoptera	Cerceris rybyensis	GCA_910591515.1_iyCerRyby1.1_genomic
Hymenoptera	Nysson spinosus	GCA_910591585.1_iyNysSpin1.1_genomic
Hymenoptera	Ectemnius continuus	GCA_910591665.1_iyEctCont1.1_genomic
Hymenoptera	Bombus terrestris	GCA_910591885.1_iyBomTerr1.1_genomic
Hymenoptera	Andrena haemorrhoa	GCA_910592295.1_iyAndHaem1.1_genomic
Hymenoptera	Ectemnius lituratus	GCA_910593735.1_iyEctLitu1.1_genomic
Hymenoptera	Dolichovespula media	GCA_911387685.1_iyDolMedi1.1_genomic
Hymenoptera	Bombus hypnorum	GCA_911387925.1_iyBomHypn1.1_genomic
Hymenoptera	Dolichovespula saxonica	GCA_911387935.1_iyDolSaxo1.1_genomic
Hymenoptera	Osmia bicornis	GCA_907164935.1_iyOsmBic2.1_genomic
Hymenoptera	Vespa velutina	GCA_912470025.1_iyVesVel2.1_genomic
Hymenoptera	Seladonia tumulorum	GCA_913789895.1_iySelTumu1.1_genomic
Hymenoptera	Sphecodes monilicornis	GCA_913789915.1_iySphMoni1.1_genomic
Hymenoptera	Tenthredo notha	GCA_914767705.1_iyTenNoth1.1_genomic
Hymenoptera	Anoplius nigerrimus	GCA_914767735.1_iyAnoNige1.1_genomic
Hymenoptera	Ancistrocerus nigricornis	GCA_916049575.1_iyAncNigr1.1_genomic
Hymenoptera	Macropis europaea	GCA_916610135.1_iyMacEuro1.1_genomic
Hymenoptera	Lasioglossum morio	GCA_916610235.1_iyLasMori1.1_genomic
Hymenoptera	Lasioglossum lativentre	GCA_916610255.1_iyLasLatv2.1_genomic
Hymenoptera	Athalia rosae	GCA_917208135.1_iyAthRosa1.1_genomic
Hymenoptera	Mimumesa dahlbomi	GCA_917499265.1_iyMimDahl1.1_genomic
Hymenoptera	Ichneumon xanthorius	GCA_917499995.1_iyIchXant1.1_genomic
Hymenoptera	Dolichovespula sylvestris	GCA_918808275.1_iyDolSylv1.1_genomic
Hymenoptera	Bombus sylvestris	GCA_911622165.1_iyBomSyle1.1_genomic
Hymenoptera	Andrena dorsata	GCA_929108735.1_iyAndDors1.1_genomic
Hymenoptera	Andrena minutula	GCA_929113495.1_iyAndMinu1.1_genomic
Hymenoptera	Bombus pratorum	GCA_930367275.1_iyBomPrat1.1_genomic
Diptera	Scaeva pyrastri	GCA_905146935.1_idScaPyra1.1_genomic
Diptera	Syritta pipiens	GCA_905187475.1_idSyrPipi1.1_genomic
Diptera	Tachina fera	GCA_905220375.1_idTacFera2.1_genomic
Diptera	Xylota sylvarum	GCA_905220385.1_idXylSylv2.1_genomic
Diptera	Eristalis tenax	GCA_905231855.1_idEriTena2.1_genomic
Diptera	Volucella inanis	GCA_907269105.1_idVolInan1.1_genomic
Diptera	Eristalis pertinax	GCA_907269125.1_idEriPert2.1_genomic
Diptera	Bibio marci	GCA_910594885.1_idBibMarc1.1_genomic
Diptera	Xanthogramma pedissequum	GCA_910595825.1_idXanPedi1.1_genomic
Diptera	Chrysotoxum bicinctum	GCA_911387755.1_idChrBici1.1_genomic
Diptera	Melanostoma mellinum	GCA_914767635.1_idMelMell2.1_genomic
Diptera	Coremacera marginata	GCA_914767935.1_idCorMarg1.1_genomic
Diptera	Thecocarcelia acutangulata	GCA_914767995.1_idTheAcut1.1_genomic
Diptera	Bellardia pandia	GCA_916048285.1_idBelPand1.1_genomic
Diptera	Platycheirus albimanus	GCA_916050605.1_idPlaAlba1.1_genomic
Diptera	Cheilosia vulpina	GCA_916610125.1_idCheVulp2.1_genomic
Diptera	Eristalis arbustorum	GCA_916610145.1_idEriArbu1.1_genomic
Diptera	Gymnosoma rotundatum	GCA_916610165.1_idGymRotn1.1_genomic
Diptera	Criorhina berberina	GCA_917880715.1_idCriBerb1.1_genomic
Diptera	Eupeodes latifasciatus	GCA_920104205.1_idEupLati1.1_genomic
Diptera	Clusia tigrina	GCA_920105625.1_idCluTigr1.1_genomic
Diptera	Sicus ferrugineus	GCA_922984085.1_idSicFerr1.1_genomic
Diptera	Sarcophaga caerulescens	GCA_927399465.1_idSarCaer1.1_genomic
Diptera	Volucella inflata	GCA_928272305.1_idVolInfl1.1_genomic
Diptera	Epistrophe grossulariae	GCA_929447395.1_idEpiGros1.1_genomic
Diptera	Myathropa florea	GCA_930367185.1_idMyaFlor2.1_genomic
Diptera	Pollenia angustigena	GCA_930367215.1_idPolAngu1.1_genomic
Diptera	Sarcophaga rosellei	GCA_930367235.1_idSarRose1.1_genomic
Diptera	Sarcophaga variegata	GCA_932273835.1_idSarVari1.1_genomic
Diptera	Leucozona laternaria	GCA_932273885.1_idLeuLate1.1_genomic
Diptera	Protocalliphora azurea	GCA_932274085.1_idProAzur1.1_genomic
Diptera	Nephrotoma flavescens	GCA_932526605.1_idNepFlae1.1_genomic
Diptera	Epicampocera succincta	GCA_932526305.1_idEpiSucc1.1_genomic
Diptera	Bombylius major	GCA_932526495.1_idBomMajo1.1_genomic
Diptera	Rhingia campestris	GCA_932526625.1_idRhiCamp1.1_genomic
Diptera	Stomorhina lunata	GCA_933228675.1_idStoLuna1.1_genomic
Diptera	Machimus atricapillus	GCA_933228815.1_idMacAtri3.1_genomic
Diptera	Cheilosia pagana	GCA_936431705.1_idChePaga1.1_genomic
Diptera	Nowickia ferox	GCA_936439885.1_idNowFero1.1_genomic
Diptera	Sarcophaga subvicina	GCA_936449025.1_idSarSubv1.1_genomic
Diptera	Thecophora atra	GCA_937620795.1_idTheAtra2.1_genomic
Diptera	Cistogaster globosa	GCA_937654795.1_idCisGlob1.1_genomic
Diptera	Bombylius discolor	GCA_939192795.1_idBomDisc1.1_genomic
Diptera	Phyto melanocephala	GCA_941918925.1_idPhyMeln1.1_genomic
Diptera	Calliphora vomitoria	GCA_942486065.1_idCalVomi1.1_genomic
Odonata	Ischnura elegans	GCA_921293095.1_ioIscEleg1.1_genomic
Odonata	Platycnemis pennipes	GCA_933228895.1_ioPlaPenn1.1_genomic
Odonata	Pantala flavescens	GCA_020796165_Panflav1_CAAS_Pfla_1.0
Psocodea	Liposcelis brunnea	GCA_023512825_ipLipBrun1
Orthoptera	Schistocerca piceifrons	GCA_021461385_ioSchPice1
Orthoptera	Schistocerca gregaria	GCA_023897955_ioSchGreg1
Orthoptera	Schistocerca americana	GCA_021461395_ioSchAmer1
Hemiptera	Aelia acuminata	GCA_911387785.1_ihAelAcum1.1_genomic
Hemiptera	Acanthosoma haemorrhoidale	GCA_930367205.1_ihAcaHaem1.1_genomic
Neuroptera	Chrysoperla carnea	GCA_905475395.1_inChrCarn1.1_genomic
Neuroptera	Chrysopa pallens	GCA_020423425_inChrPall1

## 3 Insect Hox gene clusters

### 3.1 Gene loss in insect Hox clusters

**Fig. 1:**
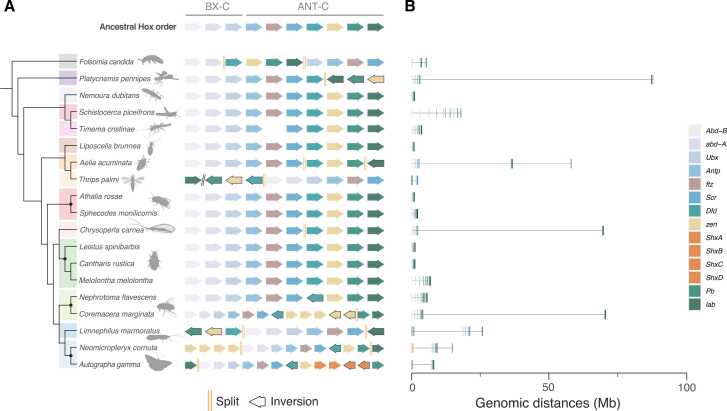
Genomic organisation and gene orientation across insect Hox clusters (A) Left shows the phylogeny for subset of species analysed. Hexapod orders, from top to bottom are: Collembola (grey), Odonata (dark purple), Plecoptera (light purple), Orthoptera (dark pink), Phasmatodea (light pink), Psocodea (brown), Hemiptera (dark orange), Thysanoptera (light orange), Hymenoptera (dark red), Neuroptera (light red), Coleoptera (dark green), Diptera (light green), Trichoptera (dark blue), and Lepidoptera (light blue).Dots on the phylogeny represent hexapod orders for which data are shown from more than one species. Right shows the order and transcriptional orientation of Hox genes (coloured as per the legend) in each species. Splits within the Hox cluster are denoted by double orange lines, inversions are annotated with a black border around the gene. Slanted double black lines represent translocation to a separate scaffold. (B) Structure of the Hox cluster per species shown using actual genomic distances. Each line represents a Hox gene as it occurs in the genome, coloured as per the legend. Genomic distances are shown in Megabases.

There are no clear examples of Hox gene loss within insects, at least for the ‘canonical’ Hox genes that play roles in specifying anteroposterior position. All eight of the expected canonical Hox genes - *pb*, *lab*, *Dfd*, *Scr*, *Antp*, *Ubx*, *abdA*, *AbdB* - are present in all insects (Fig. 1). This contrasts to some other arthropod lineages (see [26]). For example, within Crustacea the *abdA* gene is proposed to be missing in three barnacles that have been studied (*Elminius modestus*, *Trypetesa lampas* and *Sacculina carcini*) and within Chelicerata the same gene has not been found in two mites (*Archegozetes longisetosus* and *Tetranychus urticae*) and a pycnogonid (*Endeis spinosa*), although not all these surveys were based on high quality genome assemblies (see [26]).

The *ftz* gene, which evolved a role in segmental patterning in insects rather than specification of position, seems to be absent in the genome of the stick insect *Timema cristina* (order Phasmatodea; [27]; assembly tcristinae_2.1). However, since this is a finding from analysis of a single genome assembly, verification is needed. The other Hox gene with a changed function in insects, the paralogy group 3 gene *zen*, is present in most insects. Interestingly, *zen* appears to be lost completely from the genomes of two related flies, *Epicampocera succincta* and *Thecocarcelia acutangulata*, which are both within the dipteran family Tachinidae. Similar loss of *zen* may have occurred within some Chelicerata, where this gene is reported absent from the genomes of the mites *Tetranychus urticae* [28] and *Metaseiulus occidentalis* [29]. Other cases of gene loss affect more recent duplicates. For example, *zen* has undergone tandem duplication in several lineages of insects and in some cases there has been secondary loss of derived *zen* duplicates (see Section 3.4). This includes a shared loss of the *zen*-derived *ShxD* gene in all Lycaenidae butterflies.

### 3.2 Splits, rearrangements and inversions in the insect Hox cluster

Even before the molecular cloning of Hox genes, it was clear that the mutant loci giving homeotic phenotypes in *Drosophila melanogaster* were located in two distinct complexes on chromosome 3: the ANT-C and the BX-C [30], [31]. Cloning revealed the ANT-C contains from *lab* to *Antp* (of the ancestral gene order), whereas BX-C contains the genes from *Ubx* to *AbdB*, with a 9.6 Mb gap between them. Splits have also occurred, at different positions, in other *Drosophila* species [17]. In the mosquito *Anopheles gambiae*, the cluster is not split. The clear implication is that an unbroken Hox cluster is ancestral for this clade of Diptera and by implication (assuming a split cluster cannot reform into a complete cluster) ancestral for all insects, as it is for the Bilateria. In surveying the structure of Hox clusters across insects, therefore, we are not asking whether a split cluster is ancestral. Instead, we can ask whether there are particular intergenic regions where splits are more frequent evolutionarily, and conversely whether particular sets of Hox genes always stay together in evolution. Here we define intergenic regions as the genomic content between the homeobox sequences of the Hox genes, as current data do not allow us to identify the ends of every transcription unit.

Examination of 243 insect genomes reveals Hox cluster splits in many species (Fig. 1). For example, these include splits in the Hox clusters of *Platycnemis pennipes* (Odonata), *Aelia acuminata* (Hemiptera), *Chrysoperla carnea* (Neuroptera), *Coremacera marginata* (Diptera), *Limnephilus marmoratus* (Trichoptera) and in *all* Lepidoptera. In some cases, these splits lead to dramatic expansion in the overall size of the Hox cluster. For example, the *lab*, *pb* and *zen* genes in *Platycnemis pennipes* are located ∼84 Mb from the rest of the cluster. Similarly, splits between *Scr* and *Dfd* and between *pb* and *lab* in *Aelia acuminata*, resulted in genomic distances of ∼33 Mb and ∼21 Mb between these genes, respectively. In *Chrysoperla carnea* the split occurs between *Scr* and *Dfd* and results in a distance of ∼67 Mb, and in *Coremacera marginata* a distance of ∼66 Mb separates *zen* and *pb*. The cluster split in Lepidoptera lies between *lab* and the rest of the cluster, and is present in every lepidopteran species analysed (124 species) including two representatives of the most basal family, Micropterygidae (*Neomicropteryx cornuta* and *Micropterix aruncella*).

The *lab* gene is found distal to the ‘posterior’ end of the cluster in most Lepidoptera (represented in Fig. 1 by the Silver-Y moth *Autographa gamma*). This repositioning is clearly a secondary event following ‘escape’ of the Hox gene from tight linkage to other Hox genes, since in *Neomicropteryx cornuta* (in the basal family Micropterygidae) the split has occurred but the repositioning has not. The finding that the *lab* gene is also distant from the rest of the Hox cluster in Trichoptera (caddisfly) genomes suggests this split probably occurred prior to the common ancestor of Lepidoptera+Trichoptera (Amphiesmenoptera). Interestingly, relocation of *lab* to a different chromosome was also found in two mosquito species, *Aedes aegypti* and *Culex quinquefasciatus* [32].

Similar cases of translocation or inversion have occurred in Odonata, Thysanoptera and Trichoptera, after splitting of the Hox cluster. This is the case for *zen*, *pb* and *lab* split from the rest of the cluster in *Platycnemis pennipes* (Odonata), *Thrips palmi* (Thysanoptera) and *Limnephilus marmoratus* (Trichoptera). Other inversions of genes occur unrelated to splits, for example, *Dfd* is inverted in *Coremacera marginata* and *Nephrotoma flavescens* (Diptera) and *Neomicropteryx cornuta* and *Autographa gamma* (Lepidoptera) (Fig. 1).

**Fig. 2:**
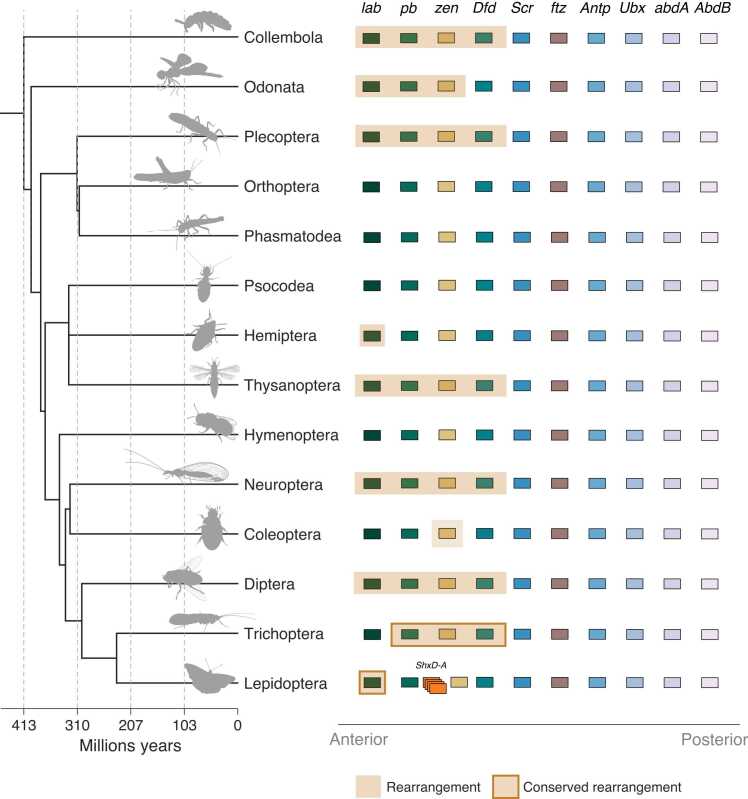
Hox genes prone to rearrangement in insect Hox clusters Left shows a time calibrated species tree of insect Orders analysed in this study. Right shows the composition of the Hox gene cluster, in their ancestral order. Shaded orange regions represent genes that have undergone rearrangement from the ancestral order of Hox genes. Those conserved across species sampled have a border around the box. Splits in the Hox cluster are not depicted.

While splits have occurred frequently in insect evolution, the overall genomic order of Hox genes in insects is comparable to that seen for the homologous genes in vertebrates. This represents the colinear correspondence between gene order and the body position where each gene is expressed and functional during early embryonic development, for those Hox genes that still play this role (Fig. 2). Although we do not find clear cases of shuffling this order when the genes are together in a single intact cluster, there are cases of rearrangement caused by cluster breakage, in some cases involving inversions. Interestingly, these changes are almost always associated with paralogy groups 1–4: *lab, pb, zen* and *Dfd* (Fig. 2). The rearrangements found affect these four Hox genes in different ways. In each case of gene, or gene block rearrangement, there is a link between splits in the Hox cluster and subsequent rearrangement events within insect orders.

In some insect orders, there are rearrangements in all species sampled; for example, five Trichoptera (caddisfly) species have *pb*, *zen* and *Dfd* in derived positions. In all four species in the family Limnephilidae, *pb*, *zen* and *Dfd* are located at the ‘posterior’ (*AbdB*) end of the cluster, with an inversion in two species, *Limnephilus marmoratus* and *Limnephilus rhombicus*. In *Eubasilissa regina* (family Phryganeidae), *pb*, *zen* and *Dfd* are found outside the cluster, upstream of *lab*. Similarly, in all Lepidoptera (butterflies and moths; 124 species) *lab* is found away from the rest of the Hox cluster. The rearrangement of *lab* away from the rest of the Hox cluster was noted previously in the silk moth *Bombyx mori* [18], [33]; the higher quality genome assemblies now available confirm that the *lab* gene is usually located at the ‘posterior’ end of the lepidopteran Hox cluster, separated by a large distance (from 1.4 Mb in *Tinea semifulvella* to 24 Mb in *Phalera bucephala*) containing numerous non-Hox genes.

In two of the three Odonata (dragonfly and damselfly) species analysed, *lab*, *pb* and *zen* are rearranged, but *Dfd* is in its ancestral position in the cluster. In the white-legged damselfly *Platycnemis pennipes* there has been an inversion that switched the order and transcriptional orientation of these genes as a block, and in the blue-tailed damselfly *Ischnura elegans* there has been an inversion plus a translocation to the other end of the cluster. In Plecoptera (stoneflies), Thysanoptera (thrips) and Neuroptera (lacewings and allies) various rearrangements are found. In certain species in these groups *lab*, *pb*, *zen* and *Dfd* have all been translocated to the ‘posterior’ end of the cluster, nearer to *AbdB,* with a subsequent inversion of this gene cassette in the plecopteran species *Nemurella pictetii*. In the only thysanopteran species in our dataset (*Thrips palmi*), an additional rearrangement resulted in *lab* positioned on a separate scaffold to the rest of the cluster and *pb*, *zen* and *Dfd* translocated to the posterior end. In Hemiptera, *lab* is located at the ‘posterior’ end of the cluster in *Acanthosoma haemorrhoidale*, although larger rearrangements affecting *lab*, *pb*, *zen* and *Dfd* in another hemipteran species (*Diaphorina citri*) has been observed [34]. Diptera displays the largest number of rearrangements, with at least five different rearrangement events occurring across the tree, resulting in translocations of one or more of the *lab*, *pb*, *zen* and *Dfd* genes to the opposite end of the gene cluster. In Coleoptera, three species show translocation of *zen* copies outside of the Hox cluster, resulting from independent lineage-specific events.

### 3.3 Hox cluster size across insects

While splits and rearrangements in the Hox cluster occur frequently across insects, there are certain genes which have rarely been split apart in the insect genomes studied to date. For example, the three genes found in the Bithorax complex of *Drosophila* (*AbdB*, *abdA* and *Ubx*) are found in the same order, in all 243 insect genomes studied, although we note that a cluster split between *Ubx* and *abdA* occurred in a clade of *Drosophila* [17]. Within the set of genes corresponding to the ANT-C of *Drosophila* (Fig. 1, Fig. 2), the genes *Antp*, *ftz* and *Scr* are most conserved in their organisation and orientation. To our knowledge, there are no known cases of split between these genes, indicating there may be a selective pressure to maintain their linkage. Indeed, overall there are relatively few cluster splits between *AbdB* and *Scr*.

**Fig. 3:**
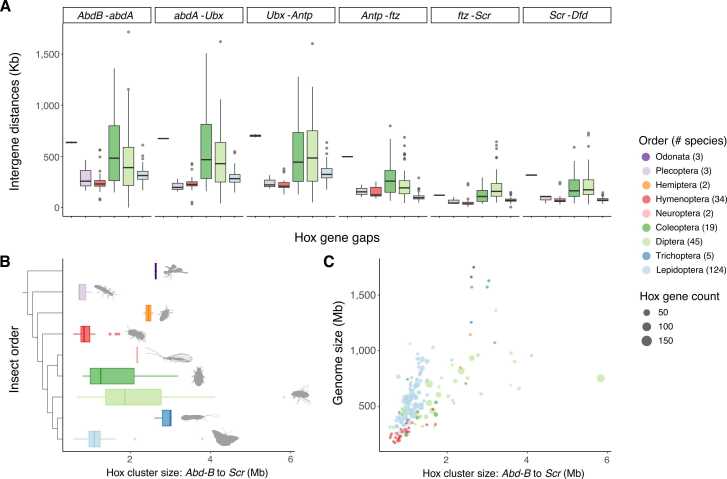
Hox cluster size across insects. (A) Distribution of intergenic regions per Hox gene in different insect orders. Only shows orders where the Hox genes are conserved in the ‘normal’ order. (B) Distribution of core Hox cluster (AbdB to Scr) size for each order, ordered by phylogeny. (C) Correlation between core Hox cluster size and genome size. Each dot represents a species, which are colour by order as per the figure legend. Dots are sized by the total number of Hox genes present in each species. In (A) and (B) boxplots have a rectangle between the 25th and 75th percentiles of the range, with the median as a dark line, whiskers reach the largest and smallest values within 1.5x Interquartile range, and outliers are points beyond 1.5 × Interquartile range (plotted using geom_boxplot in ggplot2).

When the intergenic distances between each pair of genes (measured as the distance between homeobox sequences of the Hox genes) are compared between insect orders, a very intriguing pattern emerges (Fig. 3A). Excluding the first three Hox genes located at the ‘anterior’ end of the cluster (*lab*, *pb* and *zen*), which underwent significant rearrangements in many different species, we see that the intergenic distances between the next four genes (*Antp*, *ftz*, *Scr* and *Dfd*) are consistently small. These four ‘tightly linked’ genes are all orthologues of the ANT-C genes of *Drosophila melanogaster*. In contrast, the intergenic distances between the three orthologues of the BX-C genes (*AbdB*, *abdA* and *Ubx*) are consistently larger. This trend is most easily seen when the distances are compared within an insect order, and is seen regardless of whether the insect order has more or less ‘relaxed’ organisation of the Hox cluster (Fig. 3A). This may imply that there is a deep and fundamental difference between Hox gene organisation between ANT-C and BX-C genes, dating to long before the homeotic complex split in *Drosophila*. Interestingly, the intergenic distance between *Ubx* and *Antp* in most insects (the position of the BX-C/ANT-C split in *Drosophila melanogaster*) falls into the range of the BX-C intergenic distances, even in gene clusters that are not split.

The relative conservation in gene order and organisation from *AbdB* to *Scr* across all orders provides a useful opportunity to compare the evolution of the Hox cluster size across insects. The size of this conserved core region of the Hox cluster ranges from 0.57 Mb (Common Plume moth *Emmelina monodactyla*; Lepidoptera) to 5.8 Mb (*Tachina fera*; Diptera). These genomic distances are much larger than the same region in vertebrates where whole Hox clusters are only ∼0.1 Mb [35], [36]. Odonata, Hemiptera and Trichoptera have consistently large sizes for this core cluster, reflecting large intergenic distances, while Coleoptera and Diptera each show great variation in cluster size across the order (Fig. 3B). For most insect orders, the size of the core part of the Hox cluster correlates with genome size (Fig. 3C). However, Diptera and Lepidoptera both show low correlation values (r = 0.37 and 0.27, respectively), suggesting that there are other factors driving the size of the Hox cluster other than genome size in these groups of insects.

The size of the Hox cluster in *Schistocerca piceifrons* (Orthoptera; assembly iqSchPice1.1) is significantly expanded with larger distances between all genes, relative to most other insects, suggesting relaxation of the constraints acting on the whole cluster (Fig. 1). When we compare cluster size across other *Schistocerca* species (*Schistocerca americana*; iqSchAmer2.1 and *Schistocerca gregaria*; iqSchGreg1.1), the total size of the Hox cluster ranges from 16 Mb to 17.8 Mb, and the ‘core’ Hox cluster size (*AbdB* to *Scr*) ranges from 10.8 Mb to 12.2 Mb, significantly larger than any other insect species analysed in this study (Fig. 3B). This contrasts with earlier (pre-genomic) analysis in *Schistocerca gregaria*, where the total cluster size was determined using chromosomal in situ hybridization and estimated to be at least 700Kb in length, and no longer that 2 Mb in total [11]. Although linkage in the Hox cluster in this genus has relaxed significantly, there are no rearrangements found in the order of the Hox genes within the genome.

### 3.4 Tandem duplication of insect Hox genes: *Zerknüllt* and *fushi tarazu*

**Fig. 4:**
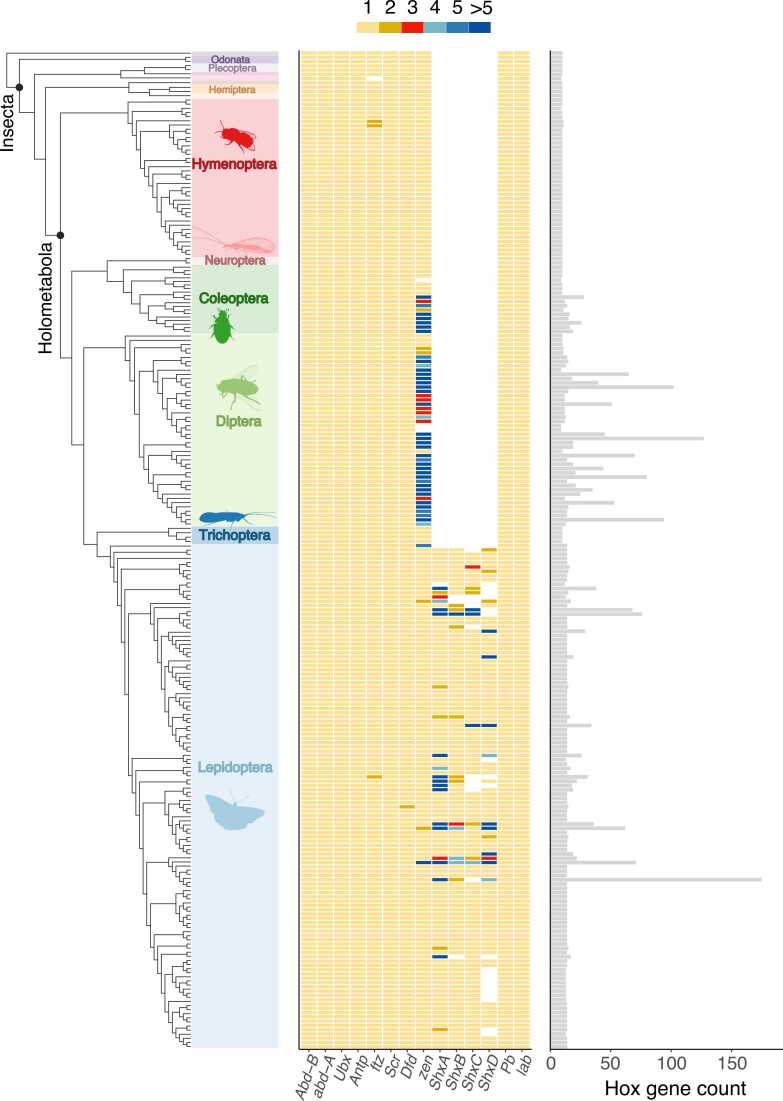
Copy number of Hox genes across insects. Left shows the phylogeny of all species analysed, with block colours signifying the Order to which they belong. Heatmap shows the copy number of all Hox genes per species. Barchart of the right shows the total number of Hox genes annotated per species.

Tandem duplication within a Hox gene cluster is rare, with some of the clearest examples being the initial expansion of the Hox cluster in early bilaterian evolution [37], [38] and expansions at the ‘posterior’ end of the cluster in vertebrates, amphioxus and echinoderms [15], [39]. In analysing publicly-available insect genomes, we find only two cases of putative tandem duplication of a canonical Hox gene (Fig. 4): two copies of *Dfd* present in *Acronicta aceris* (Sycamore Moth) and two copies of *pb* present in *Micropterix aruncella*. While genomic position and gene trees provide support for these putative Hox duplicates as real events, since they are present in a single species each of the findings needs further verification. Indeed, it is expected that tandem duplications of canonical Hox genes would be deleterious since they could disrupt the spatial regulation of these genes, and thereby disrupt anteroposterior body patterning. In contrast, the two Hox genes that have derived roles, *ftz* and *zen*, might be expected to have less constraint against duplication. This is because the *zen* gene lost its ancestral homeotic function in early insect evolution, acquiring a novel role in the formation of extraembryonic tissues, while *ftz* has a new role in segmentation.

As noted in Section 3.1, a putative loss of *ftz* is observed in one insect, and conversely there are two *ftz* copies in *Spilarctia lutea* (Buff Ermine Moth), as well as two closely related wasps: *Vespula germanica* and *Vespula vulgaris* (Fig. 4). Finding the duplication in related species gives stronger support to this observation. Duplications of *zen* in insects have been known about and intensively studied for many years. First, there is a well-studied duplication of *zen* in *Tribolium castaneum*, which gave *zen* and *zen2* [13]. Recent work has shown that this duplication is shared by three closely related *Tribolium* species, and that the gene products interact in a negative feedback loop that may confer precision of temporal expression [40]. Second, it was shown over 20 years ago that the dipteran *bcd* gene is a derived tandem duplicate of *zen* [41]. This duplication was followed by extensive sequence divergence in the locus which became *bcd*, in a classic case of ‘asymmetric sequence divergence’ where one daughter gene undergoes far more sequence change than the other [42], [43]. Key amino acid changes in the homeodomain include a mutation from glutamine to lysine at position 50 (Q50K) and a switch from methionine to arginine at position 54 (M54R); these substitutions contributed to changing downstream targets and altered biological role [44], [45]. Third, a further duplication of *zen* within the *Drosophila* genus produced *zen2*. Fourth, the most dramatic cases of *zen* duplication have been reported in Lepidoptera. In ditrysian lepidopterans, the *zen* gene duplicated to give four additional fast-evolving copies named *ShxA*, *ShxB*, *ShxC* and *ShxD* [19]. These highly derived Shx genes are expressed in the developing serosa, not the embryo itself, and may pre-pattern this extraembryonic tissue, as judged by the striking pattern of maternal RNA localisation [19]. These genes duplicated even further in *Bombyx mori* resulting in at least 12, possibly 15, tandem Shx gene copies [18], [19]. Furthermore, recent work has shown that the extreme duplication in *Bombyx* is not unique: at least 18 other lineages of Lepidoptera have highly expanded sets of Shx genes, in some cases reaching over 100 homeobox copies (Fig. 4). There has also been occasional loss of specific Shx genes; for example, *ShxD* was lost in butterflies of the family Lycaenidae (‘blues’ and their allies) and fritillary butterflies of the genus *Melitaea* [46]).

With the availability of many high-quality insect genomes, it is now possible to ask if there are additional cases of *zen* duplication, in addition to those mentioned above (Fig. 4). Within the coleopteran species for which genomes are available, multiple duplications of *zen* occur in the Cucujiformia infraorder and range from 2 copies in *Polydrusus cervinus* (weevil) to 17 copies in *Harmonia axyridis* (harlequin ladybird) and 19 copies in *Pyrochroa serraticornis* (cardinal beetle). This is in addition to the well-studied duplication in *Tribolium*. In Diptera, copy number of *zen* ranges from a single copy in the early diverging lineages to 118 in *Tachina fera* and 93 in *Sarcophaga variegata*. Even within a family of flies for which there is a large number of species sampled, Syrphidae, there is significant variation in *zen* copy number between these related species (Fig. 4). The number of copies and the branching patterns within the gene tree (Fig. 4) suggest that large tandem duplication events occurred multiple times independently in this lineage.

It is striking that duplication of *zen*, and its progenitors (Shx in Lepidoptera) occur only in the highly speciose orders Diptera, Lepidoptera and Coleoptera, within the holometabolous insects. As described above, *zen* lost its homeotic function early on in insect evolution, and in many insect species is involved in development of extraembryonic membranes. In insects these membranes consist of two distinct layers: the amnion and serosa (these form a single epithelium known as the amnioserosa in higher flies) [47], [48]. The amnion is the inner membrane which surrounds the ventral side of the developing embryo, while the serosa is an outer membrane which lies just inside the chorion and envelops the embryo, amnion and yolk [49], [50], [51]. This structure is hypothesised to be involved in a wide range of functions unrelated to development of body form, such as a general protective role including structural stability, water regulation and desiccation resistance [52], [53], [54], and innate immune response [55], [56], [57]. Interestingly, while this dual structure is present in most pterygote insects, derived hymenopterans (Apocrita) usually lack an amnion, or have a temporary amniotic-like structure which covers the yolk [58], [59]. It is intriguing to consider whether the highly dynamic copy numbers of *zen* along with its functions in the extraembryonic tissues may have played a role in facilitating speciation and adaptation to diverse habitats in Diptera, Lepidoptera and Coleoptera. Indeed, it is striking that copy number variation of *zen* is particularly variable in highly speciose families such as Syrphidae and Coccinellidae (Fig. 4). However, whether these large expansions in gene number are functional, or even expressed during early development, requires further analyses. Furthermore, the neutral theory posits that increases in copy number may not always have adaptive significance, and may instead result from mutational processes within the genome, affected by intragenomic variation in copying fidelity and the effects of transposable element accumulation.

## 4 Conclusions

We are entering a new era of genomics, as new technologies are facilitating the imminent sequencing and assembly of thousands of eukaryotic species. At the time of writing, there are more than 200 high quality, complete insect genomes available for analysis, and although this number is expected to rise very rapidly, now is an excellent time to pause and take stock of the lessons that can be learned. This is an opportune time for two reasons. First, the available high quality genomes span a wide phylogenetic diversity of insects, including representatives of at least 13 orders (Odonata, Plecoptera, Orthoptera, Phasmatodea, Psocodea, Hemiptera, Thysanoptera, Hymenoptera, Neuroptera, Coleoptera, Diptera, Trichoptera, Lepidoptera). Second, within some taxa (notably Lepidoptera and Diptera) ‘deep dives’ have been undertaken, yielding genomes from closely related species, thereby permitting insights into genomic change on shorter time frames. Here we have used these data, in combination with previously published analyses, to compare Hox gene cluster organisation across insects. We have searched for patterns of evolutionary conservation or general trends across insects, examples of convergent evolution, and anomalies.

First, we examine gene loss and conclude that canonical Hox genes have not been lost in insect evolution: *pb*, *lab*, *Dfd*, *Scr*, *Antp*, *Ubx*, *abdA*, *AbdB* are present in all insects studied. The two ‘non-canonical’ Hox genes, *zen* and *ftz*, are lost rarely. We find two closely related insect species putatively lacking *zen*, possibly a shared loss inherited from a common ancestor, and one example of a putative loss of *ftz*. The rarity of these losses highlights that further verification is needed. However, the finding that canonical Hox genes are never lost in insects has a biological implication. We suggest that each Hox gene has remained indispensable through insect radiation because segment number and tagmatization, has remained consistent, giving no opportunity for gene redundancy and loss.

Second, we find many independent cases of splitting of the insect Hox gene cluster, in an analogous fashion to the separation of ANT-C and BX-C in *Drosophila melanogaster*. Although these splits can occur in several different places in the Hox cluster, they are most commonly seen affecting the first four paralogy groups (PG1 to PG4): *lab*, *pb*, *zen*, and *Dfd*. There are cases where just *lab* (PG1) is split away (Lepidoptera and Trichoptera), one dipteran in which *lab* plus *pb* are separated away, Odonata with *lab*, *pb* and *zen* split away, and many insects with a split between *Dfd* and *Scr* (separating PG1 to PG4 from the rest). We do not find cases of complete ‘atomisation’ of the Hox cluster, as seen in larvacean chordates and predatory mites for example. It would be interesting to compare patterns of Hox cluster breakage and rearrangement with the overall genome-wide recombination and inversion rates for each taxa, to test if Hox cluster rearrangements reflect general genomic properties. From the pattern of splitting observed, we suggest that insect Hox genes are not generally regulated as a whole cluster, but there are selective pressures acting to prevent many rearrangements. These selective pressures could include shared regulation of neighbouring games, interdigitated control (enhancers for one Hox gene located beyond the neighbouring gene) or simply a high density of regulatory elements. We suggest these constraints are lowest around paralogy groups 1–4. We speculate that shared and interdigitated control may have evolved around ‘posterior’ insect Hox genes to fine-tune expression within overlapping domains in the abdomen.

Third, it has long been known that insect Hox gene clusters have much larger intergenic distances than in vertebrates. We find that intergenic distances in the Hox cluster vary greatly across insects, with particularly large genomic distances in Orthoptera, Odonata, Hemiptera and Trichoptera, and highly variable intergenic distances in Coleoptera and Diptera. Intergenic lengths correlate with genome size in most, but not all, insect orders. We note a striking and puzzling trend in intergenic distance within insect Hox clusters: the distances between ‘posterior’ genes are consistently greater than distances between each pair of ‘central’ or ‘anterior’ genes. Specifically, intergenic distances from *AbdB* to *Antp* are greater than intergenic distances across the rest of the cluster. We do not know the biological basis for this observation. Counterintuitively, the region with the largest intergenic distances is also the region least prone to genomic rearrangement in evolution. We suggest that fundamental mechanisms of gene regulation may be different at the two ends of the insect Hox gene cluster.

Fourth, we examine gene duplication and conclude that insect Hox genes are rarely duplicated, with the exception of *zen*. We do find putative cases of *Dfd* duplication and *pb* duplication, but these are seen in single genomes and require further verification. A *ftz* duplication is seen in genome assemblies for two wasps and can be treated as more definitive. The *zen* gene, in contrast, has undergone tandem duplication many times independently, undergoing dramatic copy number expansion in some insect lineages. The most striking examples of *zen* duplication are seen in genomes from the highly speciose orders, Coleoptera, Diptera and Lepidoptera, where over 100 *zen*-derived homeobox sequences can be present in some species. It is unclear why such dramatic copy number changes have occurred, and indeed whether retention of extra genes was selectively advantageous through subfunctionalization, neofunctionalization or simply dosage effects. The fact that *zen* genes play roles in extraembryonic patterning, rather than position-specific cell fate in the embryo, may underpin why tandem duplications are not instantly deleterious, but this does not seem to explain the preponderance of *zen* gene arrays observed. Further work is required to determine if the locus is particularly prone to unequal crossover at meiosis, and therefore a hotspot of mutation, and/or whether duplicated *zen* genes were repeatedly recruited to novel roles in extraembryonic membrane patterning as insects adapted to their multitude of ecological niches.

## Declarations of interest

None.
